# Functional expression of a novel α-amylase from Antarctic psychrotolerant fungus for baking industry and its magnetic immobilization

**DOI:** 10.1186/s12896-017-0343-8

**Published:** 2017-02-28

**Authors:** Lei He, Youzhi Mao, Lujia Zhang, Hualei Wang, Siti Aisyah Alias, Bei Gao, Dongzhi Wei

**Affiliations:** 1 State Key Lab of Bioreactor Engineering, New World Institute of Biotechnology, East China University of Science and Technology, P.O.B.311, 130 Meilong Road, Shanghai, 200237 China; 2 Institute of Ocean and Earth Sciences, C308 Institute of Postgraduate Studies, University of Malaya, Kuala Lumpur, 50603 Malaysia

**Keywords:** α-Amylase, Antarctic fungus, Biochemical properties, Bread quality, Immobilization

## Abstract

**Background:**

α-Amylase plays a pivotal role in a broad range of industrial processes. To meet increasing demands of biocatalytic tasks, considerable efforts have been made to isolate enzymes produced by extremophiles. However, the relevant data of α-amylases from cold-adapted fungi are still insufficient. In addition, bread quality presents a particular interest due to its high consummation. Thus developing amylases to improve textural properties could combine health benefits with good sensory properties. Furthermore, iron oxide nanoparticles provide an economical and convenient method for separation of biomacromolecules. In order to maximize the catalytic efficiency of α-amylase and support further applications, a comprehensive characterization of magnetic immobilization of α-amylase is crucial and needed.

**Results:**

A novel α-amylase (*AmyA1*) containing an open reading frame of 1482 bp was cloned from Antarctic psychrotolerant fungus *G. pannorum* and then expressed in the newly constructed *Aspergillus oryzae* system. The purified recombinant AmyA1 was approximate 52 kDa. AmyA1 was optimally active at pH 5.0 and 40 °C, and retained over 20% of maximal activity at 0–20 °C. The *K*_m_ and *V*_max_ values toward soluble starch were 2.51 mg/mL and 8.24 × 10^−2^ mg/(mL min) respectively, with specific activity of 12.8 × 10^3^ U/mg. AmyA1 presented broad substrate specificity, and the main hydrolysis products were glucose, maltose, and maltotetraose. The influence of AmyA1 on the quality of bread was further investigated. The application study shows a 26% increase in specific volume, 14.5% increase in cohesiveness and 14.1% decrease in gumminess in comparison with the control. AmyA1 was immobilized on magnetic nanoparticles and characterized. The immobilized enzyme showed improved thermostability and enhanced pH tolerance under neutral conditions. Also, magnetically immobilized AmyA1 can be easily recovered and reused for maximum utilization.

**Conclusions:**

A novel α-amylase (*AmyA1*) from Antarctic psychrotolerant fungus was cloned, heterologous expression in *Aspergillus oryzae*, and characterized. The detailed report of the enzymatic properties of AmyA1 gives new insights into fungal cold-adapted amylase. Application study showed potential value of AmyA1 in the food and starch fields. In addition, AmyA1 was immobilized on magnetic nanoparticles and characterized. The improved stability and longer service life of AmyA1 could potentially benefit industrial applications.

**Electronic supplementary material:**

The online version of this article (doi:10.1186/s12896-017-0343-8) contains supplementary material, which is available to authorized users.

## Background

α-Amylase (EC 3.2.1.1) is an endo-acting enzyme which catalyzes random hydrolysis in the interior of the starch molecules [1]. α-Amylase has broad applications in various industries including pharmaceutical, detergent, textile, paper, and of course, the food field [2–5]. In order to meet the increasing demands of biocatalytic tasks, considerable efforts have been made to isolate enzymes produced by organisms living in extreme physicochemical conditions. Although numerous mesophilic and thermophilic amylases have been well studied, cold-adapted enzymes, which possess improved catalytic efficiency at low temperatures that compensates for the inherent reduction in reaction rates, still receive little attention. Only a few bacterial cold-adapted amylases have been successfully expressed in *E. coli* [6], but there have been scant studies on enzymes from cold-adapted fungi [7].

Fungal amylases are more preferred in the food industry, especially in relation to bread and baking due to their generally recognized as safe status. Among them, cold-adapted fungal amylases are particularly used in industrial processes as energy savers. *Geomyces. pannorum,* isolated from Victoria Land (continental Antarctica), is a psychrophilic and psychrotolerant saprophytic fungus. It produces abundant cold-adapted α-amylases, indicating its significant role as a desirable candidate in food processing. However, previous studies mainly focused on strain screening and its enzyme-producing ability [8], a rare report intensively researched its biochemical properties and applications.

Several attempts have been made to improve the cost-effectiveness of fungal enzymes. Immobilization is suggested to be advantageous, since it facilitates the recovery and reuse of the expensive enzymes [9]. Recently, iron oxide nanoparticles with uniquely large surface-to-volume ratio and quantum size effects provide an economical and convenient method for immobilization and separation of biomacromolecules [10]. Fe_3_O_4_-PVA was prepared and applied in immobilization of transaminase [11]. Fe_3_O_4_–κ–carrageenan nanoparticles were produced and adhered to pullulanase [12]. Nanoparticle Fe_3_O_4_@SiO_2_–NH_2_ carrier was also developed, and alkaline pectinase was successfully coupled on it [13]. *A. oryzae* α-amylase was also magnetically immobilized, and its parameters for hydrolysis of sweet potato starch were investigated [10]. However, the comparison of free and immobilized α-amylase was not studied in detail. Thus, we tested the nanoparticles immobilization in order to compare and improve the enzymatic properties and expand the potential value of fungal α-amylase in various industrial applications.

In this study, we constructed an *A. oryzae* expression system based on the *pyrG* nutritional selection marker. Meanwhile, we reported cloning of a novel fungal, cold-adapted amylase, expressed in a newly constructed *A. oryzae* system. Here, we also examined the recombinant enzyme (AmyA1) in baking by exploring the role of AmyA1 in bread production. Furthermore, to widen application of AmyA1, we immobilized α-amylase onto magnetic nanoparticles, and investigated the detailed biochemical characters.

## Methods

### Strains, plasmids, and media

*E. coli* DH5α (Tiangen, China) was used for construction and routine propagation of plasmids. *Aspergillus oryzae* auxotrophic host (Δ*pyrG*, Δ*ligD* and Δ*pyrG*Δ*ligD*) was derivatived from the wild strain *A. oryzae* RIB40 (ATCC 42149) by gene recombination. *Geomyces pannorum* AK07KGI1001R1-2(1) (R1-2 for short, CCTCC AF2014016) was kindly donated by University of Malaya [7]. The plasmid pANE and pBC12ANH were preserved in our lab. The pBluescript II SK (+) vector was used as the skeleton for constructing expression vectors.

Potato/Dextrose/Agar (PDA) slant was used for growth of the spores of wild type and transformants. CM medium (Dextrose 1 g, NaNO_3_ 0.6 g, Peptone 0.2 g, Uridine 0.1221 g, Yeast extract 0.1 g, K_2_HPO_4_ 0.104 g, KH_2_PO_4_ 0.08 g, MgSO_4_ 0.052 g, KCl 0.05 g, Trace element 100 μL, diluted with water to 100 mL) was applied to cultivate the uracil deficient strains. MM plate (Sucrose 20.52 g, Dextrose 1 g, NaNO_3_ 0.6 g, K_2_HPO_4_ 0.104 g, KH_2_PO_4_ 0.08 g, MgSO_4_ 0.052 g, KCl 0.05 g, Trace element 100 μL, agar 1.25 g, diluted with water to 100 mL) was used as a selective medium for transformants. Culture medium added with dextrin (Dextrin 2 g, Yeast extract 0.5 g, Sucrose 0.3 g, Peptone 0.1 g, NaNO_3_ 0.1 g, MgSO_4_ · 7H_2_O 0.05 g, FeSO_4_ · 7H_2_O 0.001 g, diluted with water to 100 mL) was applied for production of α-amylase. Martin-modified medium (Pepton 0.5 g, Yeast Extract 0.2 g, K_2_HPO_4_ 0.1 g, MgSO_4_ 0.05 g, supplemented with 2 g starch as the sole carbon when necessary, diluted with water to 100 mL) was used to cultivate *G. pannorum*.

### Development of fungal transformation system

The Δ*pyrG* strain was initially derived by deleting the orotidine-5-monophosphate decarboxylase gene (*pyrG*) through targeted gene replacement [14]. Subsequently, the Δ*ligD* strain was generated by homologous recombination with the *pyrG* marker gene. Then the selected marker *pyrG* was removed by self-deleting and the Δ*pyrG*Δ*ligD* strain was obtained and used as host for further experiments [15].

A shuttle vector was constructed for expression of recombinant α-amylase in the Δ*pyrG*Δ*ligD* strain. Firstly, pBC12ANH was used as a template to obtain the elements. Fragment I (708 bp) consisting of *A. oryzae* α-amylase (TAA) promoter, signal peptide and His-tag was amplified using primers FIf/FIr with *Kpn* I and *Xho* I restriction sites respectively (Table 1). Secondly, fragment II (875 bp) including TAA terminator and fragment III (1397 bp) containing the *pyrG* of *A. nidulans* were obtained by PCR using pBC12ANH and pANE as template with primers FIIf/FIIr and FIIIf/FIIIr, respectively (Table 1). After digestion by *Kpn* I/*Xho* I, fragment I was reclaimed and connected with pBluescript II SK (+), generating shuttle vector pSKNH. Then pSKNH was digested with *Sma* I/*Xba* I. The linearized vector pSKNH, fragment II and fragment III were integrated by pEASY-Uni SeamLess Cloning And Assembly Kit (Transgen, China) to create recombinant expression vector pSKNHG, which was validated by DNA sequencing.

**Table 1:** Primers used in this study

Designation	Primer sequence (5′-3′)
FIf	CGGGGTACCGAATTCATGGTGTTTTGATC^a^
FIr	CCGCTCGAGGCTAGCATGGTGATGGTGAT
FIIf	GGGTGGAGAGTATATGATGG
FIIr	ATTCTTGAGGACCATTACTG
FIIIf	GCAACTTCCTCGAGAACGCG
FIIIr	CCCTTTTAGTCAATACCGTTAC
AmyA1f	ATGTTTTTCAACTGCCCTGC
AmyA1r	TCAAGGGCAATAGCTGCCCT
AmyA2f	CTAGCTAGCATGTTTTTCAACTGCCCTGC
AmyA2r	TCCCCCGGGTCAAGGGCAATAGCTGCCCT

^a^The sequence underlined was introduced

### Cloning of a novel α-amylase gene

DNA manipulations were carried out following the standard procedures. *G. pannorum* was incubated on the Martin-modified plate supplied with 2% soluble starch as the sole carbon source at 20 °C for 5 days. Total RNA was isolated from mycelia harvested from agar plates using Total RNA Kit I (Omega, USA). Reverse transcription was carried out according to the Primescript™ II 1st strand cDNA Synthesis Kit (TaKaRa, Japan), and the first strand cDNA was used as a template for gene amplification. Afterwards, primers AmyA1f/AmyA1r (Table 1) were synthetized in accordance with the gene sequence of the hypothetical *Geomyces* (*Pseudogymnoascus*) *pannorum* VKM F-4515 (FW-2607) α-amylase (GenBank Accession No. KFY52584.1). Sequence analysis was performed using DNAMAN software (Lynnon Biosoft) and NCBI server (http://blast.ncbi.nlm.nih.gov/Blast.cgi). The isoelectric point (pI) and molecular weight (MW) were predicted on the ExPaSy Server (http://web.expasy.org/compute_pi/). Signal peptide was analyzed by SignalP 4.1 (http://www.cbs.dtu.dk/services/SignalP/).

### Expression and purification of recombinant α-amylase

Primers AmyA2f/AmyA2r (Table 1), with *Nhe* I and *Sma* I restriction sites respectively, were synthesized to amplify the complete ORF of *AmyA1* gene. The purified PCR products were digested, reclaimed and cloned into plasmid pSKNHG, generating recombinant expression vector pSKNHG-AmyA1. Afterwards, PEG-CaCl_2_ mediated transformation was completed as previously described [16]. Transformants were selected according to their growth capability on MM plates, and then confirmed by PCR. Transformants were cultured for 6 days with an inoculum (about 5 × 10^6^ spores/50 mL) in fermentation medium at 20 °C, 200 rpm.

The supernatant was separated by vacuum filtration and subjected to a HisTrap HP column (GE Healthcare, USA) pre-equilibrated with buffer A (50 mM sodium phosphate buffer, pH 7.4, containing 0.5 M NaCl and 20 mM imidazole). Then the column was washed with 10 ml of buffer A, and 10 ml of buffer B (50 mM sodium phosphate buffer, pH 7.4, containing 0.5 M NaCl and 100 mM imidazole) in order to wash out completely the unbounded protein. Subsequently, the target protein AmyA1 was eluted with buffer C (50 mM sodium phosphate buffer, pH 7.4, containing 0.5 M NaCl and 200 mM imidazole). Eluted protein showing α-amylase activity was pooled, desalted using desalting column (GE, Healthcare, USA), and determined by enzyme assays and SDS-PAGE. Protein concentration was calculated by the Lowry method using bovine serum albumin (BSA) as the standard.

### Enzyme assays and biochemical characterization

The amylolytic activity was assayed using the DNS method [17]. The optimum pH and temperature of recombinant α-amylase (AmyA1) was determined by incubating enzyme with 1% (w/v) starch in a range of buffers (pH 2.0–9.0, 0.1 M) at 40 °C, or at different temperatures (20–70 °C) in 0.1 M citrate buffer (pH 5.0). In order to evaluate the thermostability of AmyA1, purified α-amylase was incubated at different temperatures (40–60 °C, pH 5.0). Samples were withdrawn at different time intervals and residual enzymatic activity against control was determined. The pH stability was studied by preincubating AmyA1 in various buffers ranging from pH 2.0 to 9.0 at 30 °C for 30 min. Residual activity after incubation was assayed using standard procedures.

To assess the effects of metal ions and chemical reagents on enzymatic activity, AmyA1 was preincubated with the individual reagents in 50 mM sodium phosphate buffer (pH 6.0) at 30 °C for 60 min. The residual activity was then determined in a standard assay medium. The enzyme with no metal ions was tested as the control. Additionally, the purified AmyA1 has been dialyzed at 4 °C for 12 h against 5 mM EGTA or 5 mM EDTA, respectively. The residual enzyme activity was checked with or without 5 mM calcium ions. The substrate specificity of AmyA1 was studied using soluble starch, potato starch, maize starch, glycogen, amylopectin, amylose, pullulan and maltooligosaccharides as substrates through the DNS method. The hydrolysis products arising from the action of AmyA1 on soluble starch were analyzed by HPLC. The conversion was evaluated on series 1200 HPLC system (Agilent Technologies, USA) using an NH_2_ column (Ultimate XB-NH2-3.5 μm USA), eluting with acetonitrile/water (85:15) at a flow rate of 0.8 mL/min. The average of triplicate measurements was used as each value.

The kinetic properties *K*_m_ and *V*_max_ values of the purified AmyA1 toward soluble starch were determined by Lineweaver-Burk double reciprocal plot at pH 5.0, 40 °C.

### Effects of AmyA1 on bread quality

The dough mixture was added with the enzyme preparation (AmyA1 or TAA) in a ratio of 10 U/g of dough. The flour and enzyme were manually mixed for 20 min until homogeneous dough was achieved [18]. Control dough was prepared without the addition of enzyme. Afterwards, doughs were kept for panning and proofing for 75 min at 38 °C and 85% relative humidity. Baking was performed in electric oven for 20 min at 180 °C (surface temperature) and 200 °C (bottom temperature). Subsequently, bread loaves were cooled at room temperature and packaged in polyethylene bags. The crumb texture of the loaves (hardness, springiness, cohesiveness, and gumminess) was measured by texture profile analysis (TPA) on a texture analyzer (TMS-PRO, FTC, USA) using a 35 mm flat-end plate with an aluminum compression disk (probe P/35). An acrylic cylindrical probe was used to compress the samples (2 cm thickness slices) by 40% of their original height (40 mm) at a speed of 1 mm/s, with a 30 s delay between the 1st and 2nd compression. All values are means from three independent experiments.

### Magnetic immobilization of AmyA1

Magnetic nanoparticles (IONPs) were synthesized by the solvothermal method [10]. Briefly, 0.2 M FeCl_3_ and 0.1 M FeSO_4_ were dissolved in 100 mL ultrapure water and blended together. The solution was vigorously stirred with drop wise addition of 50 mL of 10% NaOH until black precipitates were obtained. The resultant precipitates were washed a couple of times using ultrapure water and separated by magnetic decantation. Precipitates were dispersed in 50 mL Tris-HCl buffer (10 mM, pH 8.5) and ultrasonicated for 15 min, after which large precipitates were removed. α-Amylase was mixed with dried IONPs in the ratio of 1:1 in phosphate buffer (pH 4.0) and shook for 30 min. Gluteraldehyde (1%, v/v) was then added and shaken continually for 2 h. Finally, the AmyA1-IONPs were collected through magnetic decantation.

### Characterization of immobilized AmyA1

The magnetic effect of IONPs and AmyA1-IONPs was checked using a strong magnet. The aggregate morphology was analyzed with scanning electron microscopy (SEM) on a Hitachi S4800 scanning electron microscope (Hitachi, Japan). Energy dispersive spectroscopy (EDS) was carried out in an X-ray spectrometer from Falion, Japan. X-ray diffraction (XRD) identification of phases structure was studied using a Rigaku D/MAX 2550 X-ray diffractometer (Rigaku, Japan) at a wavelength of 0.154 nm.

The characteristics of magnetically immobilized AmyA1 were determined subsequently. The effect of pH on the immobilized enzyme was evaluated through pre-incubating AmyA1-IONPs at room temperature in various buffers (pH 2.0–9.0, 0.1 M) for 2 h. The residual enzymatic activity was determined as described in Enzyme assays and biochemical characterization section. The effect of temperature on the immobilized enzyme was determined by detecting the residual enzymatic activity after pre-incubating in phosphate buffer (0.1 M, pH 7.0) at temperatures ranging from 20 to 70 °C for 2 h. The reusability of the magnetically immobilized AmyA1 was investigated under constant conditions. The AmyA1-IONPs were recovered by magnetic separation after each batch reaction, and washed with PBS for latter batches. The residual activity of the immobilized enzyme after each cycle was normalized to the initial value, which was taken as 100%.

The mRNA sequence of *AmyA1* has been deposited into GenBank database under accession number **KU925863**.

## Results

### Cloning and sequence analysis of the α-amylase gene from *G. pannorum*

Based upon the genome sequence of *Geomyces* (*Pseudogymnoascus*) *pannorum* VKM F-4515 (FW-2607), the *AmyA1* gene, encoding a putative α-amylase, was obtained from *G. pannorum* 1-2. The cDNA of *AmyA1* consisted of 1482 nucleotides, corresponding to a protein of 493 amino acid residues (Additional file 1: Figure S1). Comparing the genomic DNA (analyzed from the genome sequence of *G. pannorum* in GenBank) and cDNA of *AmyA1*, two introns with lengths of 52 and 56 bp were identified, which are less than that of mesophilic α-amylases such as α-amylase (TAA) from *A. oryzae* containing 8 introns. However, the GU-AG classical rule of exon/intron splice junctions in filamentous fungi was strictly followed.

**Fig. 1:**
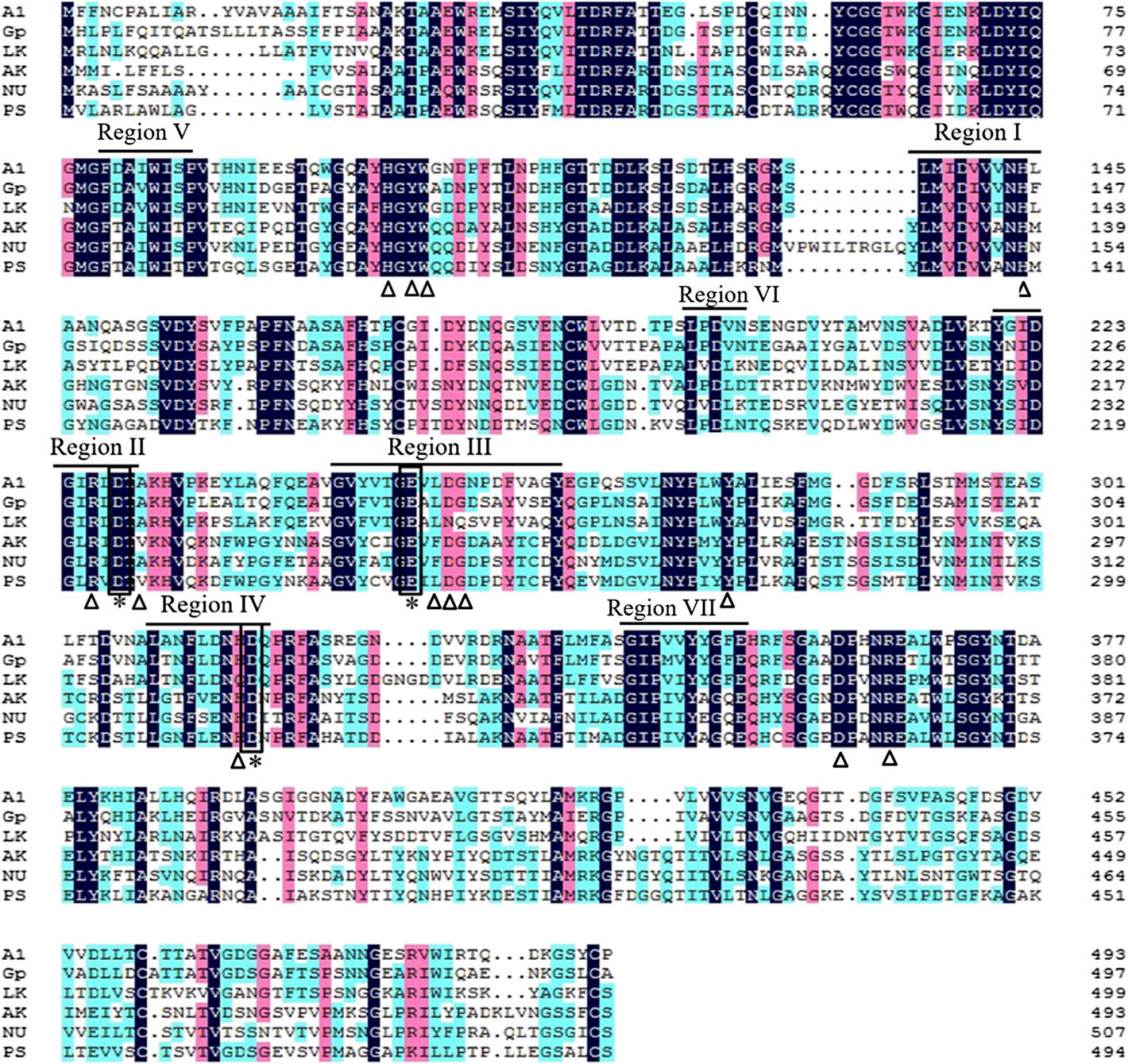
Multiple-sequence alignment of amino acids of AmyA1. The sequences of the mature proteins from *G. pannorum* (Gp, AHN65136.1), *Lipomyces kononenkoae* (LK, AAO12212.1), *Aspergillus kawachii* IFO 4308 (AK, GAA91738.1), *Neofusicoccum parvum* UCRNP2 (NU, XP_007584707.1), *Penicillium* sp. 3-5 (PS, AIF73124.1) have been aligned by introducing gaps to maximize the similarity. Regions (I, II, III, IV) are the four conserved sequence regions within α-amylase family. The structures are denoted as follows: three proposed catalytic residues (*), the potential active sites for substrate binding (△)

According to the homology search of the deduced amino acid sequences by BLAST, AmyA1 attaches to the GH 13 family of glycoside hydrolase. It shares the highest amino acid identity of 66% with that from *Geomyces pannorum* (GenBank Accession No. KFZ10929.1), and only 54 and 44% identity with the α-amylases from *Lipomyces kononenkoae* (GenBank Accession No. AAO12212.1) and *Aspergillus kawachii* (GenBank Accession No. XP_007584707.1), respectively, suggesting that AmyA1 is a novel α-amylase (Fig. 1).

Visual inspection of the alignment indicated seven common conserved regions (I-VII) associated with catalytic activity and characteristics the of α-amylase family (Fig. 1) [19]. The first 25 residues of AmyA1 encoding a potential canonical signal peptide coincides with the prediction online. The putative catalytic active site Asp228, Glu252, and Asp317 are correlated well with those in the α-amylase family. The calculated pI/Mw of the AmyA1 was estimated to be 4.48/53,607 and 4.45/50,992 for the native protein (containing signal peptide) and mature peptide, respectively.

### Expression and purification of AmyA1 in *A. oryzae*

The *A. oryzae* auxotrophic host (Δ*pyrG*Δ*ligD*) was obtained by homologous recombination and used as the host. The general vector designed for heterologous protein expression was then constructed (Additional file 2: Figure S2). The produced vector, pSKNHG, contained a complete expression cassette together with a signal peptide, His-tag and *pyrG* gene as the selectable marker. The expression vector pSKNHG harboring the *AmyA1* gene was introduced into the Δ*pyrG*Δ*ligD* strain. The positive transformants were fermented at 20 °C and a superior strain (A1-3) was selected by enzymatic activity assay. The recombinant α-amylase was purified by affinity chromatography and the purified enzyme showed a clear band of 52 kDa according to SDS-PAGE analysis (Additional file 3: Figure S3), which is similar to that of TAA (54 kDa). The specific activity of purified AmyA1 was 12.8 × 10^3^ U/mL. The kinetic parameters were evaluated, and the *K*_m_ and *V*_max_ values of purified AmyA1 were 2.51 mg/mL and 8.24 × 10^−2^ mg/(mL min) towards soluble starch, respectively.

### Biochemical characterization of AmyA1

**Fig. 2:**
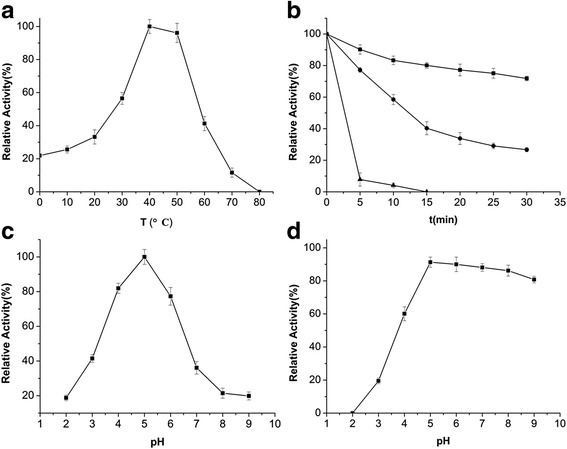
Effect of temperature and pH on purified recombinant α-amylase. **a** Temperature dependence of AmyA1; **b** Thermostability of AmyA1. Incubation at 40 °C (■), 50 °C (●), 60 °C (▲). **c** Relative activity profile of AmyA1 at different pH conditions; **d** Relative stability of AmyA1 at different pH conditions. The values shown are the means of three independent experiments and the error bars represent the standard deviations

The temperature activity and stability profiles of the purified AmyA1 are illustrated in Fig. 2a and b. The enzyme was optimally active at 40 °C and exhibited over 20% of its maximal activity at 0–20 °C. The activity declined sharply when temperature exceeded 50 °C. AmyA1 maintained over 70% of initial activity after incubation at 40 °C for more than 30 min, while losing most activity when incubated above 50 °C. The optimum pH of AmyA1 was determined over a pH range of 2.0–9.0. According to the pH-activity profile shown in Fig. 2c, maximum activity was observed at pH 5.0 and more than 70% activity was retained at pH 4.0–6.0. AmyA1 was relatively stable during treatment in neutral and basic buffers. The residual activities were nearly 80% of the control value after incubated in the pH range of 5.0–9.0 for 30 min (Fig. 2d).

The effects of different metal ions and reagents at a final concentration of 5 mM and 0.5% (v/v) on AmyA1 activity were analyzed (Table 2). The activity was slightly enhanced by Ca^2+^, Mg^2+^, and mildly decreased by EDTA, which was not consistent with previous reports that most α-amylases require calcium or magnesium for activity or stability [20]. Additionally, the catalytic efficiency of AmyA1 was negligibly effected in the presence of Zn^2+^, Cu^2+^, and slightly inhibited by Co^2+^, Mn^2+^, Fe^2+^, and isopropanol, DMSO (retaining above 80% of activity), but strongly reduced by Ni^2+^, Tween-80, Tween-20, Triton X-100, and methanol. Moreover, the activity of dialyzed AmyA1 was investigated with or without Ca^2+^. In accordance with our previous result, EDTA and EGTA did not affect the activity of AmyA1 significantly, and Ca^2+^ had mildly effect on the enzymatic activity (Table 3).

**Table 2:** Effect of different metal ions and chemical reagents on recombinant AmyA1

Metal ions/chemical reagents	Concentration (mM)	Relative activity (%)^a^
Blank	5	100 ± 3.7
Mg^2+^	5	117.1 ± 4.2
Co^2+^	5	91.1 ± 3.2
Mn^2+^	5	93.1 ± 3.1
Fe^2+^	5	95.9 ± 2.9
Cu^2+^	5	99.4 ± 5.3
Ni^2+^	5	61.3 ± 1.8
Ca^2+^	5	113.0 ± 4.8
Zn^2+^	5	100.7 ± 3.5
EDTA	5	81.7 ± 2.1
Teween-80	0.5% (v/v)	63.2 ± 1.5
Teween-20	0.5% (v/v)	45.3 ± 1.7
Triton X-100	0.5% (v/v)	58.8 ± 2.2
Methanol	0.5% (v/v)	53.2 ± 1.5
Isopropanol	0.5% (v/v)	85.0 ± 3.0
DMSO	0.5% (v/v)	81.3 ± 2.7

^a^The data are the means of three independent experiments

**Table 3:** Effect of calcium ions on recombinant AmyA1

Metal ions/chemical reagents	Concentration (mM)	Relative activity (%)^a^
Blank^b^	5	100 ± 1.0
EDTA^c^	5	73.0 ± 1.8
EGTA^d^	5	82.2 ± 3.5
Ca^2+^+ EDTA^e^	5	81.9 ± 2.7
Ca^2+^+ EGTA^f^	5	89.1 ± 3.8

^a^The data are the means of three independent experiments

^b^The activity of AmyA1 was determined in standard assay condition in the absent of metal ions

^c^The enzyme was dialyzed against 5 mM EDTA, then the activity was determined in standard assay condition

^d^The enzyme was dialyzed against 5 mM EGTA, then the activity was determined in standard assay condition

^e^The enzyme was dialyzed against 5 mM EDTA, then the activity was determined in the presence of 5 mM Ca^2+^

^f^The enzyme was dialyzed against 5 mM EGTA, then the activity was determined in the presence of 5 mM Ca^2+^

The substrate specificity of AmyA1 was ascertained using various carbohydrates at final concentration of 1% (w/v). As illustrated in Table 4, AmyA1 displayed broad substrate specificity that could efficiently hydrolyze all the tested substrates, except pullulan. The highest activity of AmyA1 was achieved on substrates maize starch and potato starch. Notably, glycogen could also be hydrolyzed effectively by AmyA1.

**Table 4:** Substrate specificity of recombinant AmyA1

Substrate (5 g/L)	Main linkage/monomer	Relative activity (%)^a^
Soluble starch	α- (1 → 4)-α- (1 → 6) glucose	100 ± 3.1
Maize starch	α- (1 → 4)-α- (1 → 6) glucose	74.1 ± 4.2
Potato starch	α- (1 → 4)-α- (1 → 6) glucose	81.3 ± 2.8
Amylose	α- (1 → 4) glucose	54.1 ± 1.9
Amylopectin	α- (1 → 4)-α- (1 → 6) glucose	69.2 ± 3.5
Glycogen	α- (1 → 4)-α- (1 → 6) glucose	46.1 ± 2.6
Pullulan	α- (1 → 6) glucose	8.2 ± 1.5
maltohexaose	α- (1 → 4) glucose	38.9 ± 3.8
maltopentaose	α- (1 → 4) glucose	30.5 ± 3.5
maltotetraose	α- (1 → 4) glucose	7.7 ± 1.7
maltotriose	α- (1 → 4) glucose	6.1 ± 1.3

^a^The data are the means of three independent experiments

**Fig. 3:**
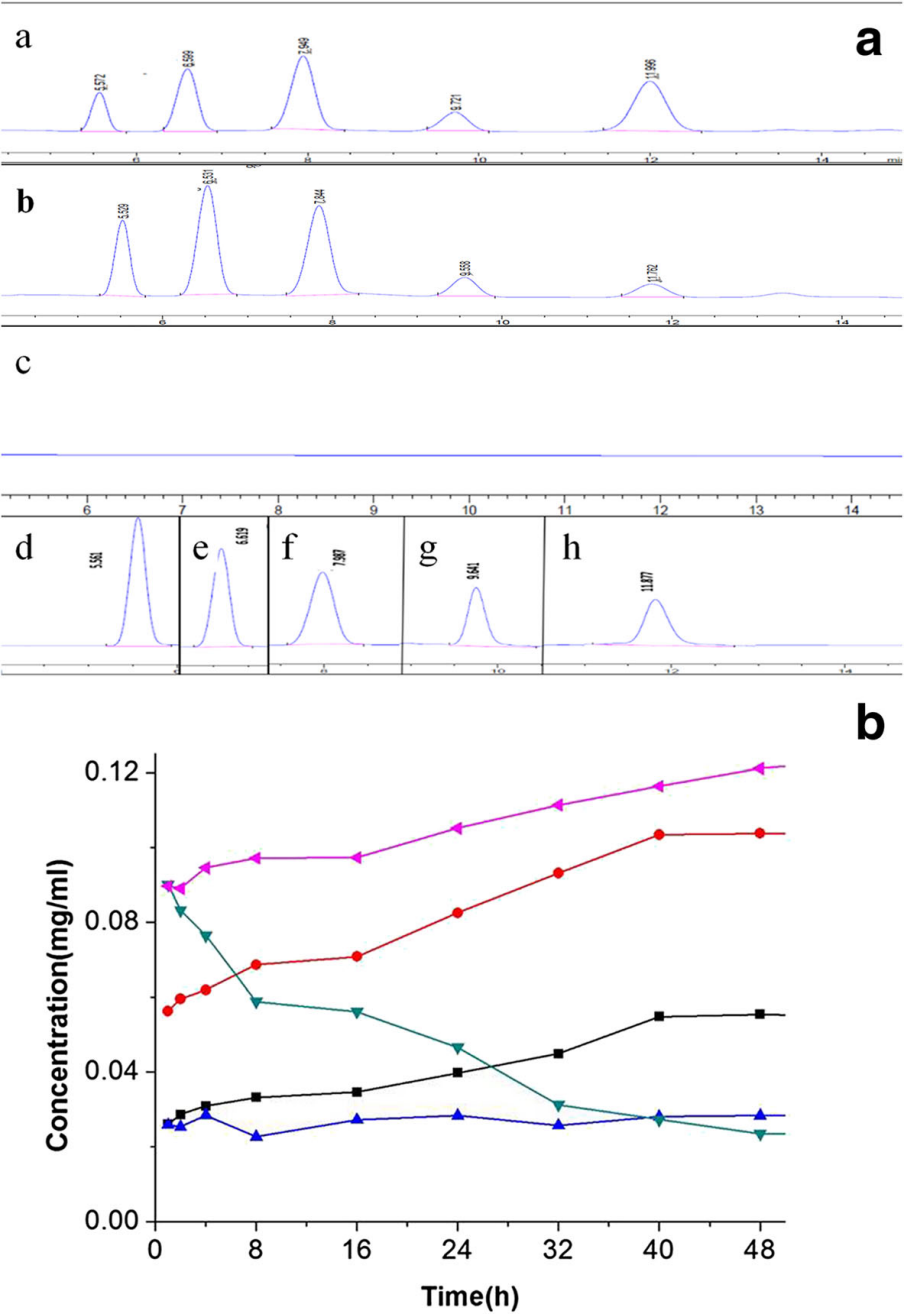
HPLC analysis of end products catalyzed by AmyA1 on soluble starch. (**a**) Reaction time: 1 h (*a*) and 48 h (*b*); Control: Reaction for 48 h without AmyA1 (*c*); Standards: glucose (*d*), maltose (*e*), maltotriose (*f*), maltotetraose (*g*), maltopentaose (*h*). The retention time of major peaks represented glucose (5.6 min), maltose (6.6 min), maltotriose (7.9 min), maltotetraose (9.6 min), maltopentaose (11.9 min) respectively; (**b**) Effect of reaction time on hydrolysis products, Abbreviations: G1 (◄), glucose; G2 (●), maltose; G3 (■), maltotriose; G4 (▲), maltotetraose, G5 (▼), maltopentaose

The hydrolyzed products were detected by HPLC with standard sugar solutions. As presented in Fig. 3a, the soluble starch was converted into glucose, maltose, maltotriose, maltotetraose and maltopentaose. During the early phase of the reaction (8 h), 19.4% (w/w) glucose, 13.5% maltose, 6.6% maltotriose, 4.5% maltotetraose and 11.7% maltopentaose were obtained, demonstrating that AmyA1 preferentially cleaves at the internal a-1,4 linkage between adjoining glucose units. As the reaction time prolonged, the amount of glucose, maltose and maltotriose increased, but the amount of maltopentaose decreased. Interestingly, the amount of maltotriose almost stayed the same (Fig. 3b). The highest hydrolysis yield of 86.4% was achieved at 48 h, and the soluble starch was catalyzed into glucose, maltose and maltotetraose accounting for 36.4, 31.3 and 16.7% respectively. Subsequently, different substrates were tested to confirm the type of amylases of AmyA1. The catalytic rate enhanced according to the increase in substrate length, indicating a higher affinity for longer substrates. Besides, HPLC analysis illustrated that AmyA1 could hydrolyze different polysaccharides and maltooligosaccharides into glucose, maltose, maltotriose, and small amounts of maltotetraose and maltopentaose (Table 5). These data confirmed that AmyA1 was an α-amylase.

**Table 5:** Product specificity profile of AmyA1^a^

Substrate (1%, w/w)	End hydrolysis products (48 h)
Amylose	G1 G2 G3 G4 G5
Amylopectin	G1 G2 G3 G4 G5
maltohexaose	G1 G2 G3 G4
maltopentaose	G1 G2 G3
maltotetraose	G1 G2 G3
maltotriose	G1 G2

^a^ *Abbreviations*: *G1* glucose, *G2* maltose, *G3* maltotriose, *G4* maltotetraose, *G5* maltopentaose

### Effects of AmyA1 on bread

**Fig. 4:**
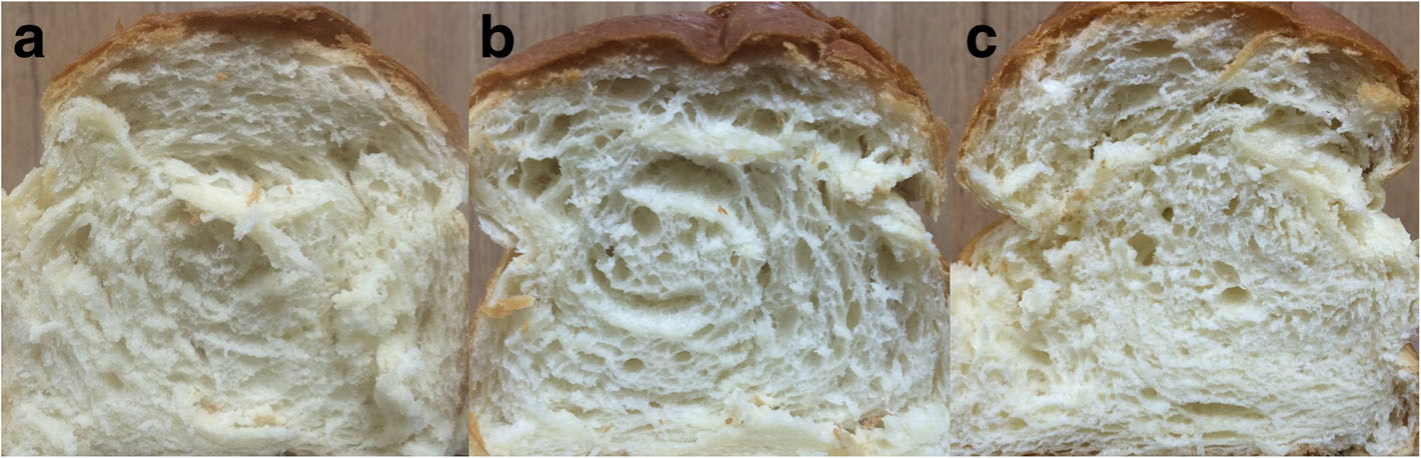
Crumb structure of the loaf supplemented with: control (without enzyme) (**a**); AmyA1 (**b**); α-amylase TAA from *A. oryzae* (**c**)

Acidic amylases, which could produce fermentable sugars and dextrin for further use by yeast, are applied in the baking industry to improve bread quality. Supplementation of AmyA1 and TAA had a significant effect on bread, which is clearly visible in the bread slices (Fig. 4). In comparison with the control, addition of AmyA1 and TAA improved bread specific volume of 26 and 18.6%, respectively. Besides, crumb structure was more uniform with homogeneous small cells when adding AmyA1. The quality of bread was illustrated directly by TPA evaluation (Table 6). These mechanical characteristics are correlated well with the sensory perception of bread and can define bread quality [18, 21, 22]. Table 6 shows that significant changes were observed in all the tested parameters of bread: the hardness and springiness increased notably, the cohesiveness increased by 14.5%, and the gumminess decreased by 14.1% as compared with the control. In particular, the effect was more pronounced with AmyA1 than that of TAA, indicating the potential value of AmyA1 in the food and starch fields.

**Table 6:** Difference in TPA supplemented with TAA and AmyA1

Substrate	Hardness (gf)	Springiness	Gumminess (gf)	Cohesiveness
Control	1314.09 ± 15.62^a^	0.812 ± 0.016	792.23 ± 11.52	0.228 ± 0.011
TAA	1439.31 ± 17.49	0.839 ± 0.013	793.00 ± 17.38	0.252 ± 0.013
AmyA1	1588.44 ± 17.55	0.837 ± 0.016	681.03 ± 15.86	0.111 ± 0.006

^a^Values are mean ± standard deviation of triplicates data

### Magnetic immobilization and characterization of AmyA1

**Fig. 5:**
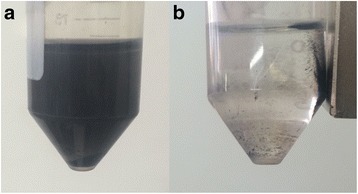
**a** AmyA1 immobilized on pretreated IONPs; **b** AmyA1-IONPs under magnetic field

Magnetic separation of the AmyA1-IONPs aggregates was achieved by placing a magnet adjacent to a vial, in which the immobilized enzyme was suspended (Fig. 5). The AmyA1-IONPs were completely accumulated near to the magnet within 5 s, and the solution became clear and transparent, demonstrating the simple and rapid method to effectively collect aggregated immobilized enzyme and reuse. In addition, removal of the magnet followed by agitation resulted in resuspension of the aggregates.

**Fig. 6:**
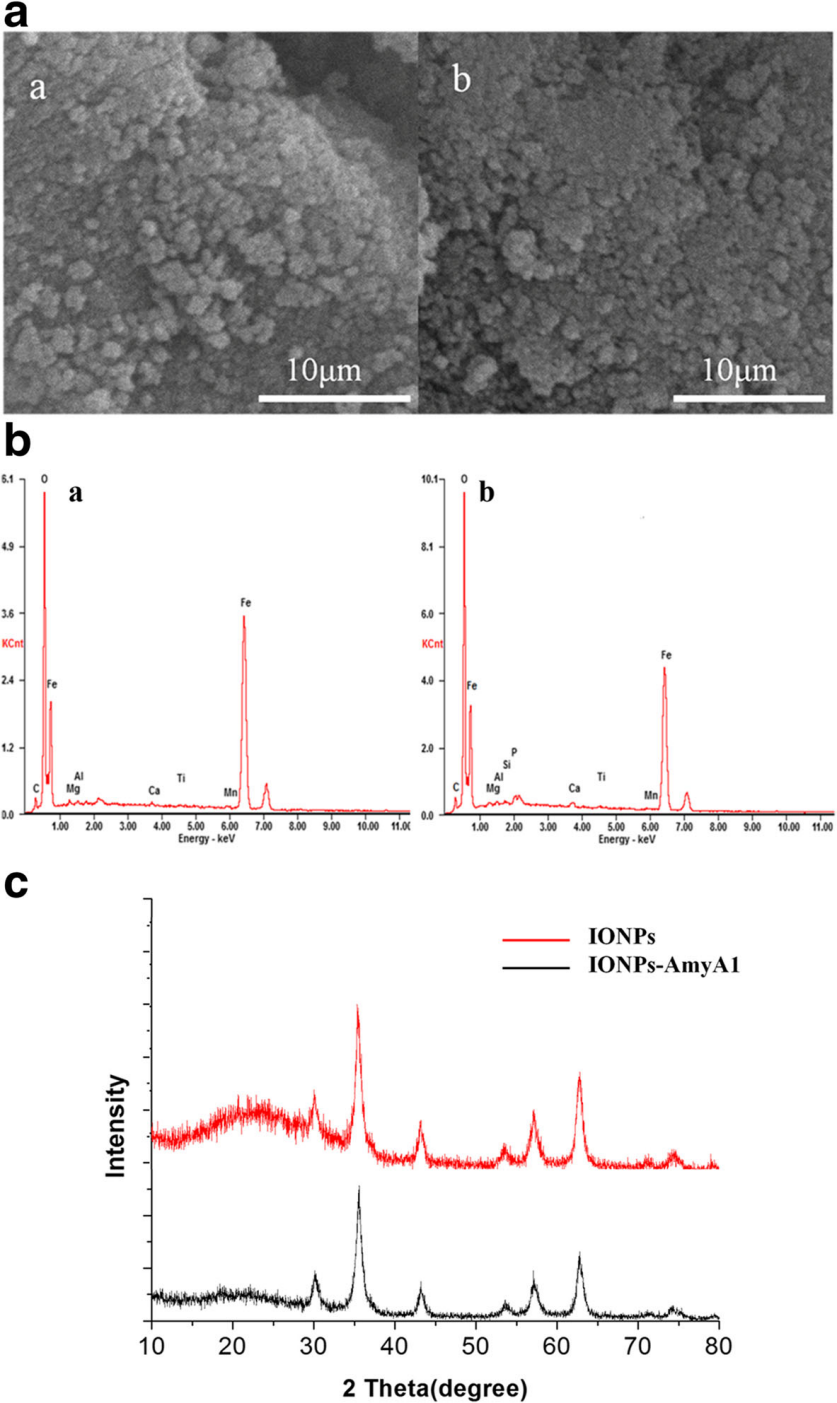
(**a**) SEM analysis of IONPs and AmyA1-IONPs (*a*: IONPs, *b*: AmyA1-IONPs); (**b**) EDS analysis of IONPs and AmyA1-IONPs (*a*: IONPs, *b*: AmyA1-IONPs); (**c**) XRD analysis of IONPs and AmyA1-IONPs

The surface characteristics of magnetically immobilized AmyA1 were investigated by SEM (Fig. 6a). Most of the magnetite particles obtained have a nearly spherical shape and uniform size. The diameter of the spheres was generally under 100 nm. The surface of AmyA1-IONPs was found to be porous in nature, which might reinforce the catalytic activity of α-amylase. Results of EDS analysis illustrated the elemental composition of AmyA1-IONPs as shown in Fig. 6B. The presence of carbon, oxygen and iron was confirmed, proving the magnetic effect of the nanocomposite of AmyA1. The identification of material phases was studied by XRD analysis (Fig. 6c). Six high peaks in the diffraction angles of 30.1°, 37.1°, 43.1°, 53.4°, 57.0°, and 62.6°, mirrored typical Fe_3_O_4_ crystal plane (220, 311, 400, 422, 511, and 440. JCPDS file, No. 85-1436), demonstrating the pure inversed spinel Fe_3_O_4_ and crystalline AmyA1-IONPs in structure. The average diameter of the crystal plane was about 40 nm, which was calculated by Debye-Scherrer formular [23].

**Fig. 7:**
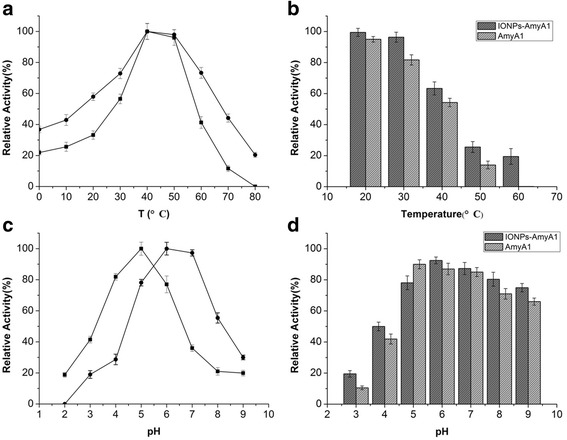
**a** Temperature dependence of AmyA1 (■) and AmyA1-IONPs (●); **b** Thermostability of AmyA1 and AmyA1-IONPs; **c** Relative activity of AmyA1 (■) and AmyA1-IONPs (●) at different pH conditions; **d** pH stability of AmyA1 and AmyA1-IONPs

The effect of temperature on the activity and stability of free and immobilized AmyA1 was investigated. Both free and AmyA1-IONPs had an optimal temperature of 40 °C. However, the immobilized enzyme showed comparatively higher activity when the temperature increased or decreased. The AmyA1-IONPs kept more than 40% of the optimal activity at 20 and 70 °C, whereas the free AmyA1 maintained only 23.2 and 11.6%, respectively (Fig. 7a). The stability of free and immobilized AmyA1 at various temperatures suggested that AmyA1-IONPs were obviously more stable than free AmyA1 after being incubated at temperatures ranging 20–60 °C for 2 h (Fig. 7b). The magnetic immobilized α-amylase retained nearly 20% of the optimal activity at 60 °C, but the free AmyA1 lost its activity within 15 min. The effect of pH was also investigated. The immobilized AmyA1 exhibited higher activity in neutral and basic conditions, with an optimal pH of 6.0–7.0, which is higher than that of free AmyA1 (pH 5.0) (Fig. 7c). Meanwhile, AmyA1-IONPs displayed better pH tolerance and maintained its vitality slightly better (Fig. 7d).

**Fig. 8:**
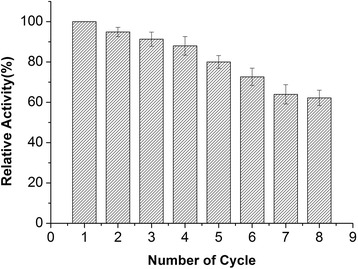
Reusability of immobilized AmyA1

The activity of AmyA1-IONPs was 11.9 × 10^3^ U/mL while the activity of free enzyme was found to be 12.8 × 10^3^ U/mL. The immobilized AmyA1 retained 93.1% of its original activity as compared to free enzyme. The α-amylase loading on IONPs was found to be 190.9 mg/g. To investigate the reusability, AmyA1-IONPs were recovered using magnetic separation after each batch reaction and reused for hydrolysis of soluble starch. The magnetic immobilized α-amylase maintained more than 90% activity in the first 3 cycles, and 60% of its initial hydrolytic activity even after 8 cycles (Fig. 8).

## Discussion

Alpha-amylase, one of the most versatile enzymes, holds a large market share with applications in a broad range of industrial processes. Diverse α-amylases from various genera have already been cloned and well characterized. To date, cold-adapted amylases from bacteria have drawn attention, such as thermolabile α-amylase from *Pseudoalteromonas arctica* and cold-adapted α-amylase from *Alteromonas haloplanctis* [24, 25]. However, the amylases from psychrophilic fungi have been poorly investigated at the molecular level. *G. pannorum*, a psychrotolerant fungus thriving in Antarctica, secretes many kinds of hydrolytic enzymes, like amylase, protease, cellulase, and esterase. In this work, a cold-adapted amylase gene *AmyA1* was successfully cloned from *G. pannorum* R1-2. Sequence analysis showed that AmyA1 shares relatively low homology with α-amylase from other genera, revealing its research significance as a novel enzyme.

Note that no enzymatic activity was detected when AmyA1 was expressed in *E. coli* and *Pichia pastoris*. However, AmyA1 was functionally heterologous expressed in *A. oryzae* and efficiently secreted into the medium, demonstrating that the *A. oryzae* system we constructed has distinct advantages for fungal genes expression. Subsequently, the AmyA1 was purified for enzymatic properties exploration. Similar to fungal α-amylases from *A. oryzae*, *A. niger*, and *Rhizopus oryzae*, AmyA1 exhibited the highest activity at neutral environment (pH 5.0–7.0) with a sharp peak in pH curve. Generally, lower optimum temperature and greater thermosensitivity than most reported fungal amylases indicate broad potential in industrial applications, such as additives in processed food, waste-water treatment, cold-washing detergents, cold-climate bioremediation, molecular-biological applications. The optimum temperature of AmyA1 is slightly lower than that of other α-amylases, and its thermolabile feature (unstable above 40 °C) are consistent with the cold-adapted amylases [24, 25], thus providing an opportunity of AmyA1 for further use. It is worth mentioning that the wild strain *G. pannorum* R1-2 could not present α-amylase activity above 30 °C, but the AmyA1 showed its optimum temperature at 40–50 °C, indicating the abundant enzyme reserves of microorganism, and its powerful resistance to harsh environment.

Generally, the main products of starch catalyzed by fungal α-amylase were maltose with a small amount of glucose [26, 27]. However, our study revealed a high-degradation rate and high-glucose product catalyzed by AmyA1, which could simplify the syrup industry by eliminating the saccharification process. Meanwhile, AmyA1 also produced abundant maltotriose as well as certain amounts of maltotetraose and maltopentaose, suggesting that AmyA1 could serve as a potential candidate for functional oligosaccharide production. In addition, substrate specificity experiments demonstrated that AmyA1 had broad substrate scope and could act on diverse kinds of starch. Note that AmyA1 could catalyze glycogen effectively, which is distinct from other α-amylases [28].

Nowadays, bread quality received a particular attention on account of its high consumption. Thus considerable efforts have given rise to improve breads that combine health benefits with good sensory properties. For instant, addition of glycogen branching enzyme led to bread volume increase, while adding esterase resulted in a decreased bread volume [22, 29]. The water and sucrose SRC values of flour reduced with the increasing level of endoxylanases [30]. Amylase action and properties also significantly affect starch structure in bread. The supplementation of *Bacillus subtilis* α-amylase caused an increase in hardness, gumminess, and a decrease in springiness, cohesiveness [18]. In comparison, addition of AmyA1 led to decrease in gumminess and increase in springiness and cohesiveness, which favor better bread quality. Furthermore, lower optimum temperature, greater thermosensitivity, and its neutral optimal pH indicate that AmyA1 would be more favored in the baking industry or related applications, because its characteristics coincide well with the condition of dough fermentation [22].

Easy separation and repeated reuse perform significant roles in utilization of enzymes. Recently, a convenient and economical method using iron oxide nanoparticles is thriving for separation of biomacromolecules [10, 31]. Several successful applications on proteins as well as cells were reported [32–34]. In order to expand the potential value of this enzyme in other areas, the purified fungal α-amylase AmyA1 was efficiently immobilized on magnetic nanoparticles and the characteristics of immobilized AmyA1 were investigated. The AmyA1-IONPs displayed more stability than free AmyA1, mainly due to the fact that IONPs made the conformation of α-amylase more rigid and lead to less denaturation of protein. Moreover, fixing the enzyme on a matrix could also decrease the protein autolysis [23]. Compared with the free enzyme, the immobilized enzyme exhibited better pH tolerance and stability under neutral conditions. This notable shift of optimum pH may be owing to the difference between H^+^ and OH^−^ concentrations in the microenvironment, which is formed by the electrostatic interactions with the carrier [35]. Meanwhile, the binding interaction between IONPs and AmyA1 could reduce drastic conformational changes during pH shifting [36]. The magnetically immobilized AmyA1 can be effectively separated, recovered and reused for maximum utilization. The immobilized α-amylase showed good stability over cycles and 60% of its initial hydrolytic activity was retained after the eighth cycle. The favorable stability and longer service life of the magnetically immobilized AmyA1 provide an opportunity for large-scale application and cost reduction.

## Conclusions

The presented study described the gene cloning, expression, purification, and characterization of a novel α-amylase from Antarctic mycelial fungus *G. pannorum*. The detailed report of the enzymatic properties of AmyA1 gives new insights into fungal cold-adapted amylase. Moreover, different application prospects of α-amylase are investigated. Application study showed the potential value of AmyA1 in traditional baking and food industry. By the means of magnetic immobilization, the improved thermostability and favorable reusability of AmyA1 could potentially support industrial production.

## Additional files

Additional file 1: Figure S1. Nucleotide and deduced amino-acid sequence of the cDNA of *AmyA1.* The bases of lowercase interrupting the coding region are introns (Intron I, Intron II). (TIF 7270 kb)
Additional file 2: Figure S2. Scheme for construction of the vector pSKNHG for α-amylase expression in *Aspergillus* sp. (TIF 12,481 kb)
Additional file 3: Figure S3. SDS-PAGE analysis of AmyA1. *Lane 1*, protein molecular mass markers; *Lane 2*, cultured supernatant of transformant A1-3; *Lane 3-6*, NPI 20, 50, 200, 500 elution of cultured supernatant of transformant A1-3; *Lane 7-8*, the purified recombinant AmyA1. (TIF 1590 kb)
