# Supplementary figures and images for: Functional expression of a novel α-amylase from Antarctic psychrotolerant fungus for baking industry and its magnetic immobilization
Source: BMC Biotechnol. 2017 Feb 28;17:22. doi: 10.1186/s12896-017-0343-8 (PMC5331696; doi:10.1186/s12896-017-0343-8)

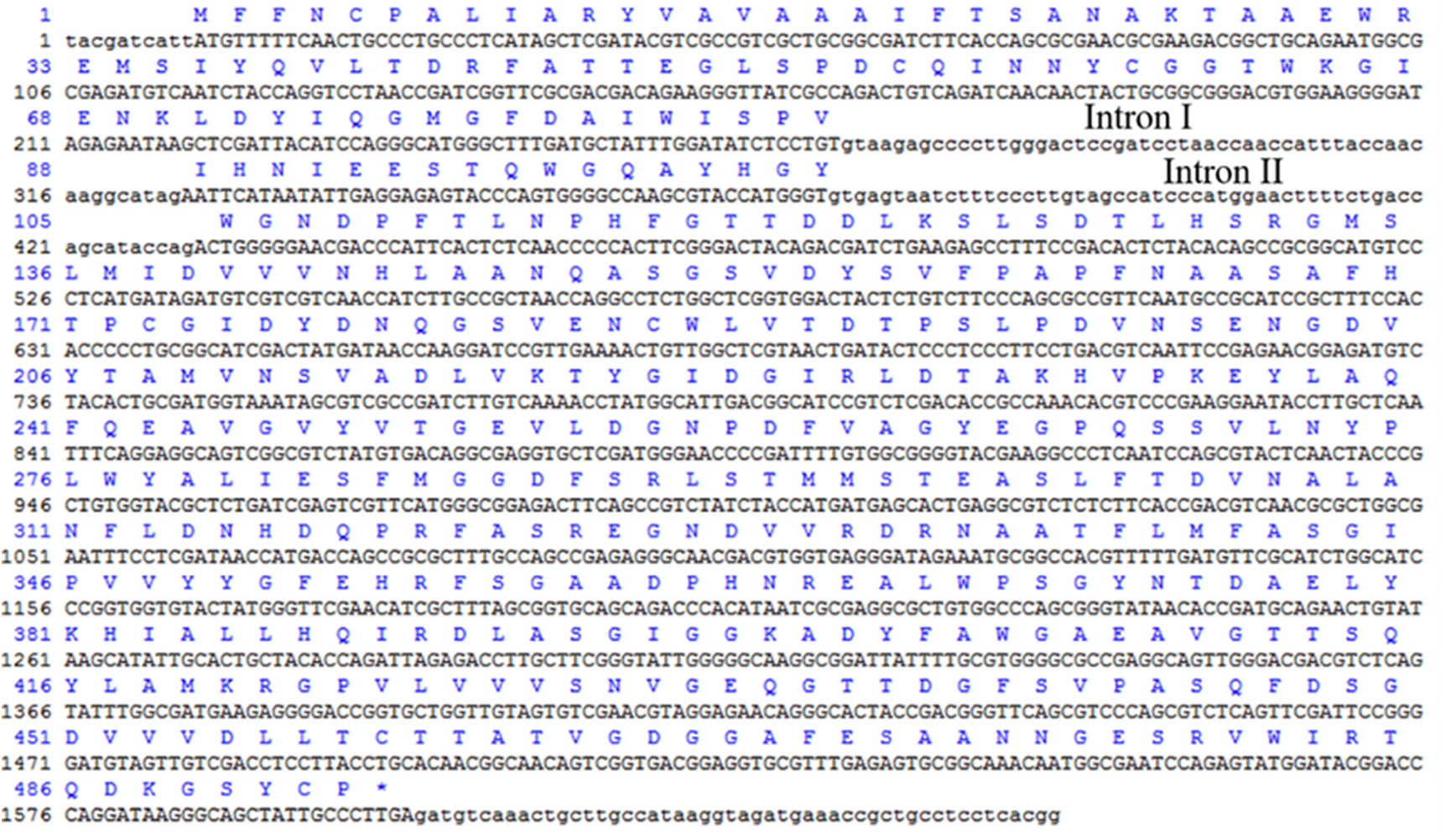

Supplement: Additional file 1: Figure S1. — Nucleotide and deduced amino-acid sequence of the cDNA of AmyA1. The bases of lowercase interrupting the coding region are introns (Intron I, Intron II). (TIF 7270 kb) [file 12896_2017_343_MOESM1_ESM.tif]

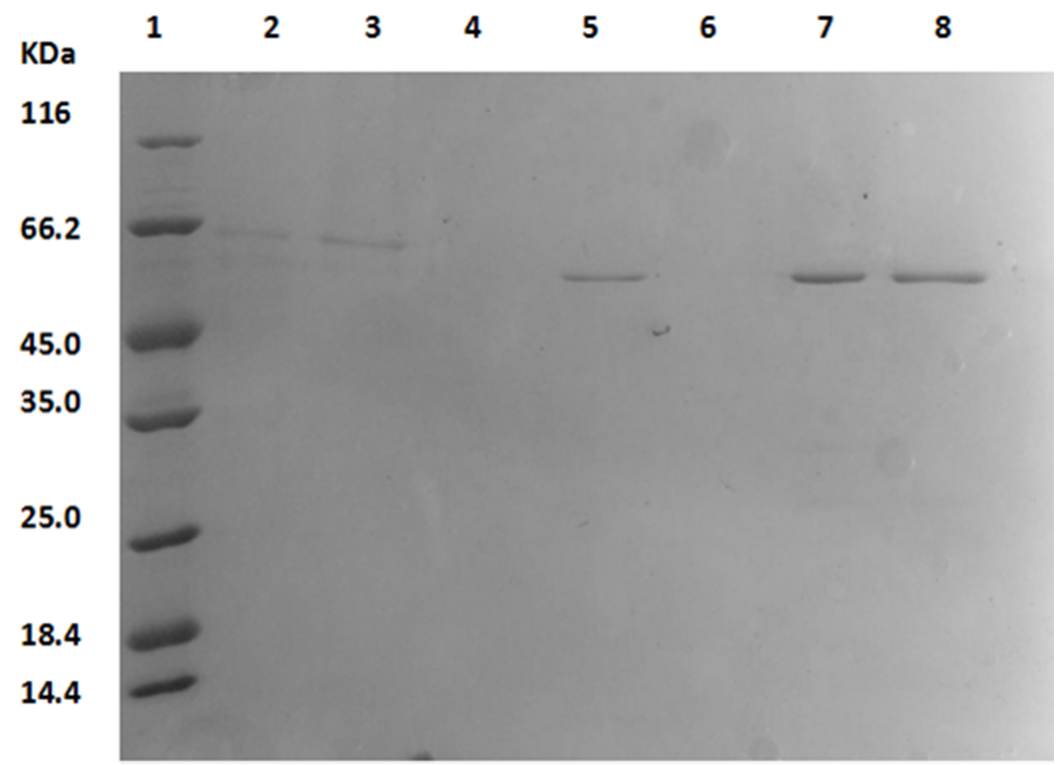

Supplement: Additional file 3: Figure S3. — SDS-PAGE analysis of AmyA1. Lane 1, protein molecular mass markers; Lane 2, cultured supernatant of transformant A1-3; Lane 3-6, NPI 20, 50, 200, 500 elution of cultured supernatant of transformant A1-3; Lane 7-8, the purified recombinant AmyA1. (TIF 1590 kb) [file 12896_2017_343_MOESM3_ESM.tif]
